# Radiomics for the non-invasive prediction of PD-L1 expression in patients with brain metastases secondary to non-small cell lung cancer 

**DOI:** 10.1007/s11060-023-04367-7

**Published:** 2023-06-29

**Authors:** Anna-Katharina Meißner, Robin Gutsche, Norbert Galldiks, Martin Kocher, Stephanie T. Jünger, Marie-Lisa Eich, Lucia Nogova, Tommaso Araceli, Nils Ole Schmidt, Maximilian I. Ruge, Roland Goldbrunner, Martin Proescholdt, Stefan Grau, Philipp Lohmann

**Affiliations:** 1 Department of General Neurosurgery, Center for Neurosurgery, Faculty of Medicine and University Hospital Cologne, University of Cologne, 50937 Cologne, Germany; 2 Institute of Neuroscience and Medicine (INM-3, -4), Research Center Juelich, Juelich, Germany; 3 Department of Neurology, Faculty of Medicine and University Hospital Cologne, University of Cologne, Cologne, Germany; 4 Center for Integrated Oncology (CIO), Cologne and Duesseldorf, Universities of Aachen, Cologne, Bonn Germany; 5 Department of Stereotactic and Functional Neurosurgery, Center for Neurosurgery, Faculty of Medicine and University Hospital Cologne, University of Cologne, Cologne, Germany; 6 Department of Pathology, Faculty of Medicine and University Hospital Cologne, University of Cologne, Cologne, Germany; 7 Department I of Internal Medicine, Faculty of Medicine and University Hospital Cologne, University Hospital Cologne, Cologne, Germany; 8 Department of Neurosurgery, University Hospital Regensburg, Regensburg, Germany; 9 Department of Neurosurgery, Klinikum Fulda, Academic Hospital of the University of Marburg, Marburg, Germany

**Keywords:** Machine learning, Artificial intelligence (AI), Radiogenomics, MRI, Brain tumors, NSCLC

## Abstract

**Background:**

The expression level of the programmed cell death ligand 1 (PD-L1) appears to be a predictor for response to immunotherapy using checkpoint inhibitors in patients with non-small cell lung cancer (NSCLC). As differences in terms of PD-L1 expression levels in the extracranial primary tumor and the brain metastases may occur, a reliable method for the non-invasive assessment of the intracranial PD-L1 expression is, therefore of clinical value. Here, we evaluated the potential of radiomics for a non-invasive prediction of PD-L1 expression in patients with brain metastases secondary to NSCLC.

**Patients and methods:**

Fifty-three NSCLC patients with brain metastases from two academic neuro-oncological centers (group 1, n = 36 patients; group 2, n = 17 patients) underwent tumor resection with a subsequent immunohistochemical evaluation of the PD-L1 expression. Brain metastases were manually segmented on preoperative T1-weighted contrast-enhanced MRI. Group 1 was used for model training and validation, group 2 for model testing. After image pre-processing and radiomics feature extraction, a test-retest analysis was performed to identify robust features prior to feature selection. The radiomics model was trained and validated using random stratified cross-validation. Finally, the best-performing radiomics model was applied to the test data. Diagnostic performance was evaluated using receiver operating characteristic (ROC) analyses.

**Results:**

An intracranial PD-L1 expression (i.e., staining of at least 1% or more of tumor cells) was present in 18 of 36 patients (50%) in group 1, and 7 of 17 patients (41%) in group 2. Univariate analysis identified the contrast-enhancing tumor volume as a significant predictor for PD-L1 expression (area under the ROC curve (AUC), 0.77). A random forest classifier using a four-parameter radiomics signature, including tumor volume, yielded an AUC of 0.83 ± 0.18 in the training data (group 1), and an AUC of 0.84 in the external test data (group 2).

**Conclusion:**

The developed radiomics classifiers allows for a non-invasive assessment of the intracranial PD-L1 expression in patients with brain metastases secondary to NSCLC with high accuracy.

## Introduction

Lung cancer accounts for up to 40–60% of brain metastases [1] and about 40–70% of patients with non-small cell lung cancer (NSCLC) develop brain metastases during the course of the disease [2, 3]. The advent of targeted therapies and immunotherapy using immune checkpoint blockade yielded considerable intracranial response rates and significantly improved the survival of patients [1]. An elevated level of the immune checkpoint protein programmed death ligand 1 (PD-L1) on tumor and immune cells may lead to increased immune evasion and tumor progression [4]. Since the expression level of PD-L1 is an important predictor for response to immunotherapy using checkpoint inhibitors. Pembrolizumab, a humanized monoclonal antibody against programmed death 1 (PD-1), is suggested as first-line therapy in NSCLC patients lacking a targetable driver gene mutation and a PD-L1 expression rate of 50% or more [1, 5, 6] because it prevents PD-1 from engaging PD-L1, which is an important predictor for response to immunotherapy using checkpoint inhibitors [7, 8]. In addition, an improved response rate of immunotherapy with checkpoint inhibitors was shown for patients with PD-L1 levels ranging from 1–49% [9, 10] and also for patients lacking any significant PD-L1 expression [11, 12]. Nevertheless, recent studies indicated heterogeneity in terms of PD-L1 expression levels in the extracranial NSCLC and brain metastases, resulting in a potentially inaccurate stratification of patients to checkpoint inhibitor immunotherapy [13, 14, 15, 16]. Therefore, a priori assessment of PD-L1 expression levels is recommended [1, 17].

Since a considerable number of NSCLC patients with brain metastases are predominantly treated by radiosurgery, tissue samples obtained from tumor resection or biopsy are generally not available. Thus, methods for a reliable non-invasive prediction of the intracranial PD-L1 expression are of high clinical relevance.

Radiomics, a method from the field of artificial intelligence, aims to extract additional information from routinely acquired imaging, usually not accessible by conventional image analysis [18, 19]. Radiomics has already demonstrated its potential in a variety of neuro-oncology studies including the histomolecular characterization of lung cancer brain metastases [20, 21, 22, 23]. Here, we evaluated the potential of anatomical MRI radiomics for a non-invasive prediction of PD-L1 expression in patients with brain metastases secondary to NSCLC.

## Patients and methods

### Ethics statement

The present study was conducted according to the guidelines of the Declaration of Helsinki, and the retrospective analysis of data was approved by the Ethics Committees of the University Hospital Cologne, Germany (approval number 19-1686) and the University Hospital Regensburg, Germany (approval number 19-1546-101).

## Patients

From 2014–2020, we retrospectively identified patients with non-small cell lung cancer brain metastases from the neuro-oncologic centers of the University Hospitals Cologne and Regensburg, Germany, who (i) had no previous local treatment, (ii) underwent preoperative contrast-enhanced MRI, (iii) had intrametastatic PD-L1 expression based on the immunohistochemical result of tissue samples after surgical brain metastasis resection.

Clinical data were obtained from electronic databases and patient files. We recorded gender, age, systemic medical therapy, time from first diagnosis to the development of brain metastases, number, size and localization of brain metastases, clinical symptoms, preoperative karnofsky performance status (KPS), histological subtype and the presence or absence of PD-L1 expression of the extracranial tumor and the brain metastases.

### Immunohistochemical analysis of PD-L1 expression

All samples sent for routine pathological analyses were evaluated for PD-L1 expression at the time of diagnosis. Formalin-fixed paraffin-embedded tissue samples were stained using primary antibody E1L3N11 (Cell Signaling Technology, Cambridge, UK) on an automated staining system with a polymer-based detection kit and DAB-chromogen (Leica Bond Polymer Refine; Leica Biosystems, Wetzlar, Germany). PD-L1 expression on tumor cells was quantified applying the Cologne score as described previously [24]. In this study, the presence of PD-L1 expression was defined by staining of 1% or more of tumor cells (Cologne Score of at least 1).

### MRI imaging

Standard preoperative structural MR imaging procedures at both University Hospitals included T1-weighted contrast-enhanced sequences for further analysis. Images were acquired during clinical routine with different scanners and imaging parameters.

### Image preprocessing and definition of tumor mask

Image preprocessing was performed as described previously [25]. In brief, after brain extraction, inhomogeneities in the MR images were corrected with a bias field correction followed by standardization of the image intensity values. Reference values for standardization were calculated from the whole brain, with tumor volumes discarded. Manual segmentations of the contrast-enhancing tumor volume were performed by an experienced neurosurgeon using ITK-SNAP and subsequently checked independently by two raters.

#### Radiomics feature extraction

Radiomics feature extraction was performed using the open-source package PyRadiomics (version 3.0.1) in Python [26]. Prior to feature extraction, images were resampled to 1 mm^3^ voxel size and discretized to a bin width of 0.15. Three basic groups of radiomics features were extracted from the segmented contrast-enhancing tumor volume, including 16 shape, 19 first order and 75 second order features derived from the underlying gray level matrices, i.e., gray level co-occurrence matrix (GLCM), gray level dependence matrix (GLDM), gray level run length matrix (GLRLM), gray level size zone matrix (GLSZM), and neighboring gray tone difference matrix (NGTDM). Features were calculated on the original image and after applying wavelet and Laplacian of Gaussian (LoG) filters, resulting in a total of 1316 radiomics features.

#### Test-retest analysis and feature selection

To avoid the usage of non-robust radiomics features, we followed the conceptual framework proposed by Zwanenburg and colleagues [27], using the image perturbation method chain that included translation, noise and volume adaption to produce an augmented version of the original image. In a test-retest approach, radiomics features were calculated and compared for both images. Repeatability between features was evaluated by the intraclass correlation coefficient (ICC). Features were considered repeatable if the lower and upper limits of the ICC 95% confidence interval were in the range of 0.91–1.00. The ICC analysis was implemented in Python (Pingouin, version 0.3.9) [28]. Following this analysis, feature correlation was assessed by the Pearson correlation coefficient. Features were considered uncorrelated if the Pearson correlation coefficient was below 0.9 otherwise only a single representative feature was used for further analysis. In total, 100 repeatable and uncorrelated features were identified.

### Model development

The development of different classification models was performed on the training data only (group 1, University Hospital Cologne). All radiomics features were standardized by subtracting the mean and dividing by the standard deviation of the training data.

The training data was divided into five randomly stratified training and validation sets, each using 70% of the data for training and 30% for validation. Each dataset maintained a similar distribution of patients across PD-L1 subtypes as the original training set. The best hyperparameters for the classifiers identified in each training set were evaluated in each corresponding validation set. Then, a feature selection was conducted by averaging the feature importance rankings across all five splits.

The process of random stratified cross-validation was repeated using only highly important features and continued until no further improvement in the average validation metric. Afterwards, the model with the best-performing features and hyperparameters was retrained on the complete training data.

### Model testing

**Fig. 1:**
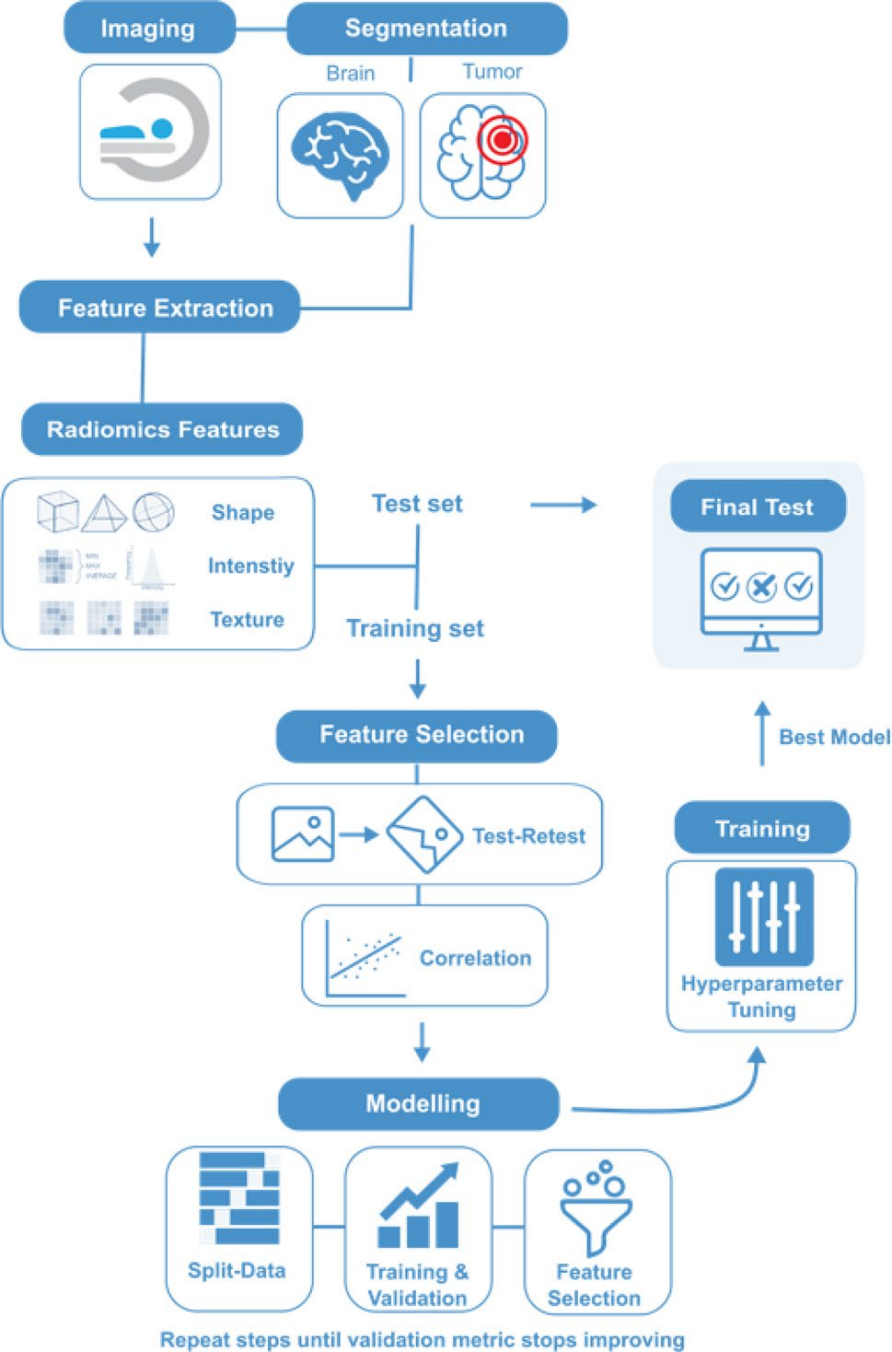
Radiomics workflow

Following model generation, the best-performing model was applied to the external test data (group 2, University Hospital Regensburg). Importantly, the final model testing was performed blinded to the PD-L1 expression rate. Afterwards, the classification results were transferred to the University Hospital Regensburg and the diagnostic performance of the classifier was assessed fully independent of the researchers involved in model generation. The radiomics workflow is presented in Fig. 1.

The prediction of the PD-L1 expression rate in patients with NSCLC brain metastases was derived from three individual models. Model 1 was a binary classifier with a discrimination threshold evaluated by a receiver operating characteristic (ROC) curve based on the contrast-enhancing tumor volume. Model 2 was trained on radiomics features only, while Model 3 was trained on a combination of the contrast-enhancing MRI tumor volume and radiomics features. Both models utilized a random forest classifier that showed the highest performance compared to logistic regression and support vector machines. All processing steps were implemented in Python (sklearn, version 0.24.1).

### Feature map extraction

To create feature maps, voxel-based feature extraction was performed using the default settings of PyRadiomics yielding local feature expression for 3 × 3 × 3 kernels. Representative images were chosen based on the maximum tumor diameter in transversal direction.

### Statistical analysis

Descriptive statistics are provided as mean and standard deviation or median and range. The diagnostic performance of each classifier was evaluated by ROC analysis and accuracy (ACC). To avoid experimental bias, the statistical analysis of the external test data (group 2, University Hospital Regensburg) was performed by an independent researcher not involved in model generation. Statistical differences in feature values between the presence and absence of PD-L1 expression were assessed by the two-tailed Mann–Whitney-U-test. P values of 0.05 or less were considered statistically significant. Statistical analyses were implemented in Python (Pingouin, version 0.3.9) [28].

## Results

### Patient and clinical characteristics

Fifty-three patients from two University Hospitals were retrospectively identified, thereof 36 patients from the Department of General Neurosurgery of the University Hospital of Cologne, Germany (mean age 62 ± 8 years; age range 47–87 years; 22 females, 14 males). Presence or absence of PD-L1 expression was equally distributed (n = 18 patients with PD-L1 expression and n = 18 patients without PD-L1 expression). Five patients (14%) showed a discrepancy of the PD-L1 expression between the extracranial NSCLC and the surgically treated brain metastasis.

Seventeen patients with surgically resected NSCLC brain metastases were identified at the Department for Neurosurgery of the University Hospital Regensburg, Germany (mean age 59 ± 12 years; age range 26–75 years; 6 females, 11 males). Seven of 17 patients (41%) had intracranial PD-L1 expression. Discordance of extra- and intracranial PD-L1 expression was present in 3 patients (18%).

The contrast-enhancing tumor volume on T1-weighted MRI was identified as a prognostic clinical parameter for PD-L1 expression in the univariate analysis. Contrast enhancement of brain metastases without PD-L1 expression was significantly larger than in brain metastases with PD-L1 expression in both datasets (group 1: 31.5 ± 26.7 mL vs. 17.8 ± 30.6 mL; p < 0.01; group 2: 20.2 ± 24.1 mL vs. 3.1 ± 3.2 mL; p < 0.01). No statistically significant differences were found in the patient’s sex, age, and KPS (all p > 0.05). Patient characteristics are summarized in Table 1.

**Table 1:** Patient characteristics

	Training set—Cologne (n = 36)			Test set—Regensburg (n = 17)		
	Total	Presence of PD-L1 expression	Absence of PD-L1 expression	Total	Presence of PD-L1 expression	Absence of PD-L1 expression
Number of patients	36	18	18	17	7	10
Sex (female/male)	22/14	7/11	15/3	6/11	3/4	3/7
Age in years at surgery (mean ± SD)	62 ± 8	62 ± 8	62 ± 8	59 ± 12	59 ± 12	59 ± 12
Number of patients with						
singular	18	10	8	6	3	3
2–3	14	8	6	9	4	5
more than 3 metastases	4	0	4	2	0	2
Median preoperative Karnofsky performance score (range)	80 (40–100)	80 (40–100)	80 (40–100)	80 (40–100)	80 (70–100)	80 (40–90)
T1-CE volume in mL (mean ± sd)	24.6 ± 29.1	17.8 ± 30.6	31.5 ± 26.7	13.1 ± 20.1	3.1 ± 3.2	20.2 ± 24.1
Time in months to detection of brain metastases (mean ± SD)	8 ± 14	8 ± 14	8 ± 14	10.6 ± 9.9	10.6 ± 9.9	10.6 ± 9.9
Histological subtype						
Adenocarcinoma	32	18	14	17	9	8
Squamous cell carcinoma	1	0	1	0	0	0
Not otherwise specified	3	0	3	0	0	0
Systemic therapy (multiple possible)						
Chemotherapy	7	3	4	4	2	2
Immunotherapy	9	3	6	2	2	0
Targeted therapy	2	0	2	2	1	1

### Classification results

**Fig. 2:**
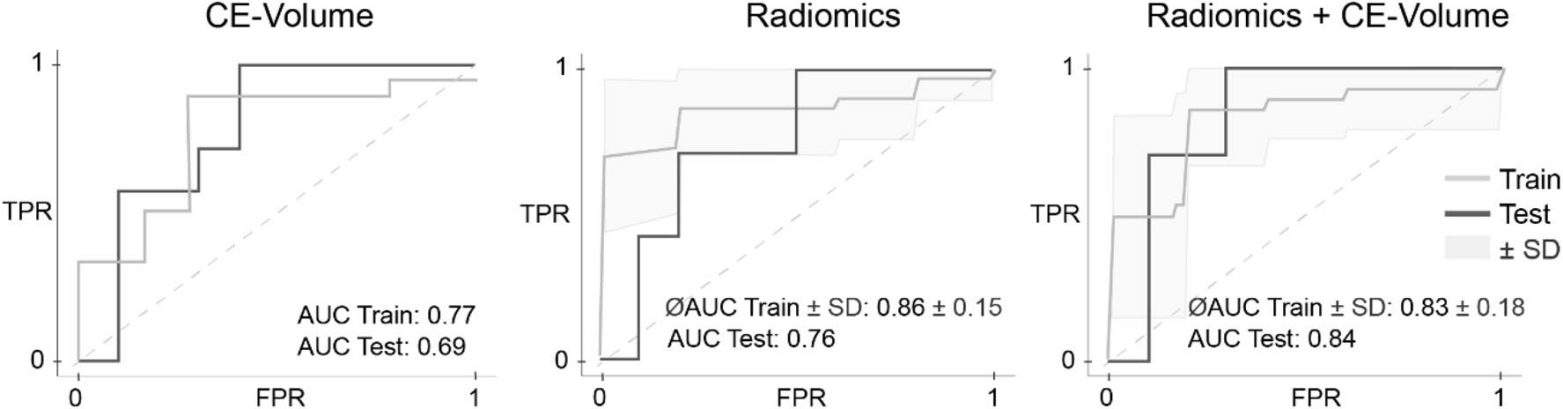
Receiver operating characteristic curves of the developed classifiers in the training and the test dataset. Left: ROC classifier using the contrast enhancing tumor volume. Center: Radiomics model based on a radiomics signature with features extracted from T1-weighted contrast-enhanced MRI. Right: Radiomics models based on a combination of the contrast enhancing tumor volume and radiomics features

We compared three models in their ability to predict the PD-L1 expression in patients with brain metastases secondary to NSCLC. A binary classifier based on the contrast-enhancing tumor volume on MRI predicted patients below 20.2 mL to have PD-L1 expression with an area under the ROC (AUC) of 0.77 in the training set, and an AUC of 0.64 in the test set (Fig. 2).

In contrast, the random forest model based on a radiomics signature of three most important features calculated on T1-weighted contrast-enhanced MRI showed a mean AUC of 0.86 ± 0.15 in the training set (Fig 2, Table 2) and an AUC of 0.76 in the test set (Fig 2, Table 2). The best diagnostic performance was achieved by combining the radiomics features and the contrast-enhancing MRI tumor volume, with an AUC of 0.83 ± 0.18 (ACC of 85%) in the training set. This performance could be confirmed in the external test dataset with an AUC of 0.84 and an accuracy of 82% (Fig 2, Table 2). The hyperparameters of the best random forest model were as follows: number of trees, 500; function to measure the quality of a split, “gini”; maximum depth of the tree, 3; minimum number of samples required to split an internal node, 2; weights associated with classes, “balanced”; all other parameters were set to default.

**Table 2:** **Top** - Results from random stratified cross validation of model 1 (contrast enhancing tumor volume) and model 2 (contrast enhancing tumor volume + radiomics features) using the data set from the University Hospital Cologne. **Bottom** - Test results of model 1 and model 2 using the independent data set from the University Hospital Regensburg.

Training								
	Radiomics model				Radiomics model + CE-volume			
CV fold	Acc [%]	AUC	Sens [%]	Spec [%]	Acc [%]	AUC	Sens [%]	Spec [%]
1	100	1.00	100.0	100	91	0.97	100	83
2	73	0.67	67	80	64	0.53	67	60
3	82	0.77	67	100	91	0.90	83	100
4	100	1.00	100	100	91	0.93	100	80
5	91	0.88	100	80	91	0.80	100	80
Mean ± SD	89 ± 12	0.86 ± 0.15	87 ± 18	92 ± 11	85 ± 12	0.83 ± 0.18	90 ± 14	81 ± 14

Test								
	Radiomics Model				Radiomics Model + CE-Volume			
	Acc [%]	AUC	Sens [%]	Spec [%]	Acc [%]	AUC	Sens [%]	Spec [%]
	77	0.76	71	80	82	0.84	100	70

Acc: accuracy; AUC: area under the receiver operating characteristic curve; CE: contrast enhancing; CV: cross validation; SD: standard deviation; Sens: sensitivity; Spec: specificity; T1c: contrast-enhanced T1-weighted MRI; T2: T2-weighted MRI

### Feature importance

**Fig. 3:**
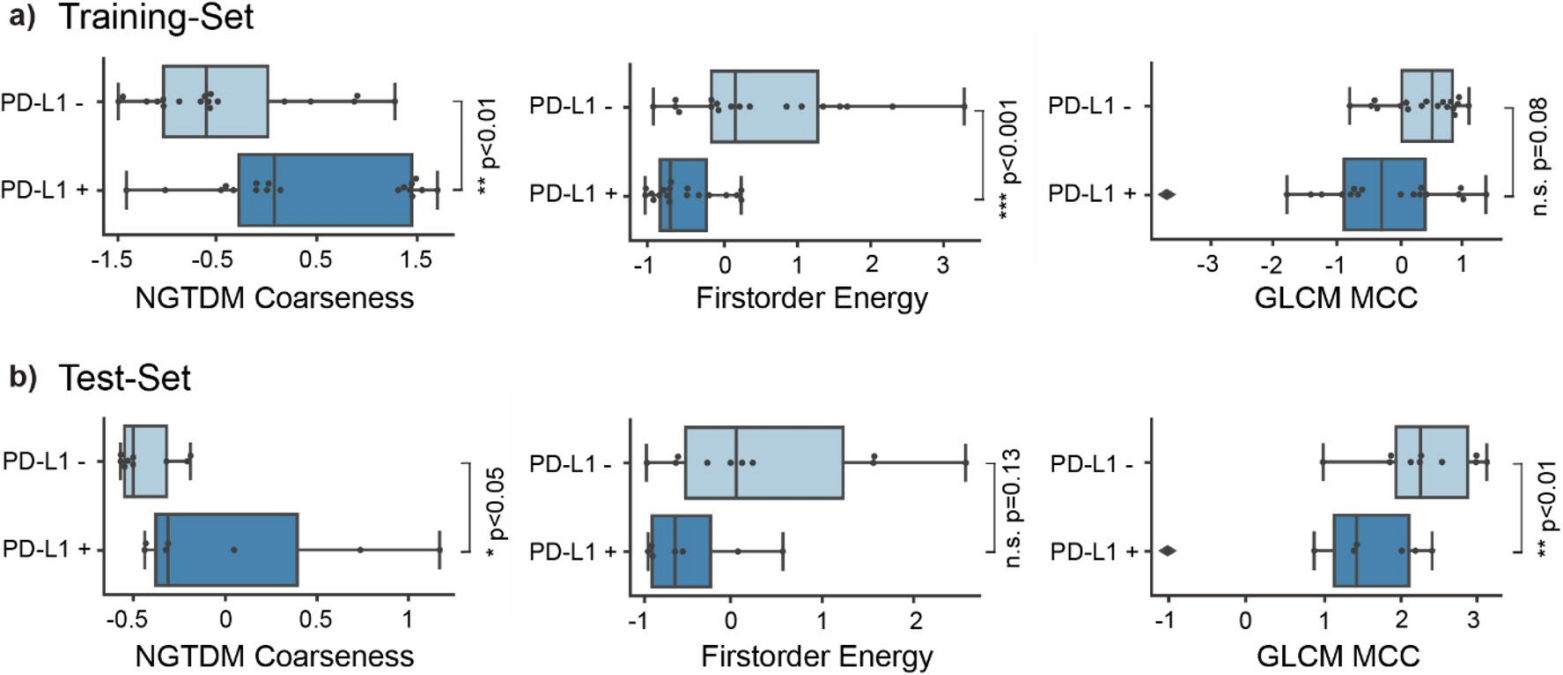
Most important features of the developed radiomics signature showing significant differences in brain metastases with present or absent PD-L1 expression in the training set **a** and the test set **b**. The developed radiomics signature included one histogram feature (i.e., energy) and two textural features (i.e., neighboring gray tone difference matrix coarseness (NGTDM coarseness) and gray level co-occurrence matrix maximum correlation coefficient (GLCM MCC).

**Fig. 4:**
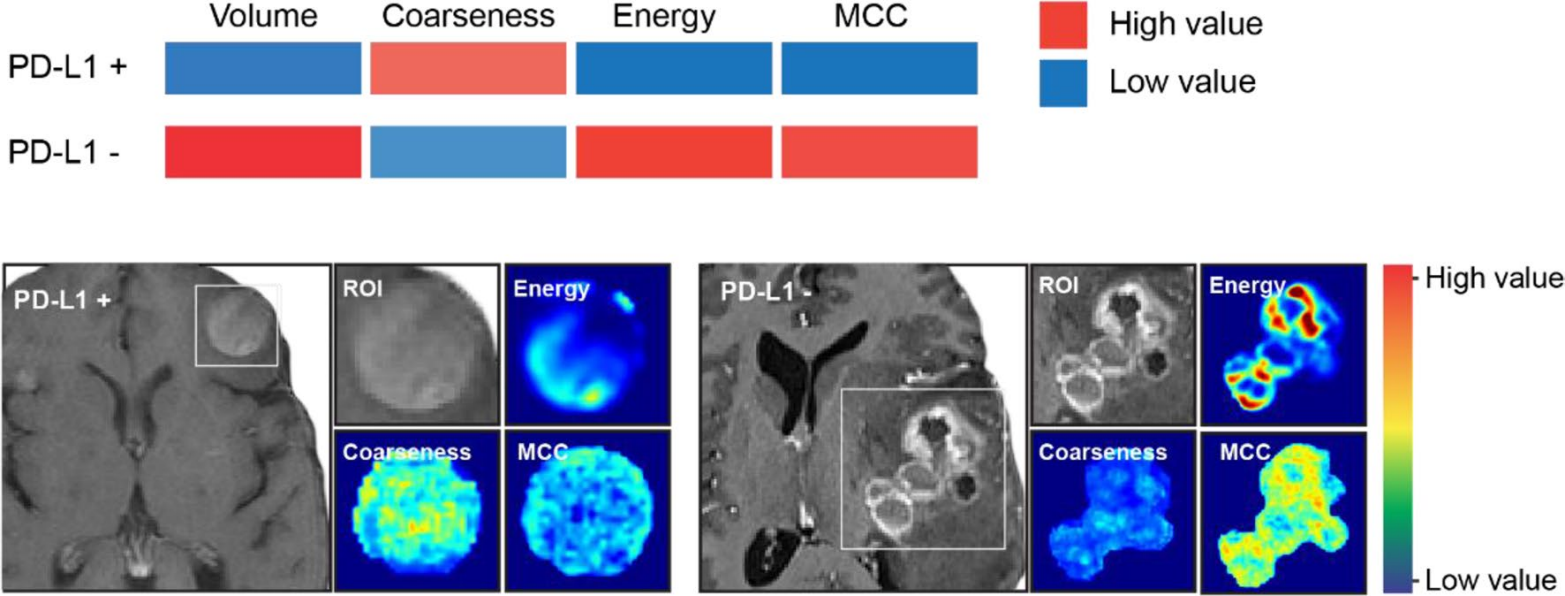
Representative feature expression maps showing distinct patterns of radiomics features that differ between the contrast-enhanced T1-weighted MRI of a brain metastasis with PD-L1 expression (left) and without PD-L1 expression (right). Brain metastases with PD-L1 expression showed a more homogenous texture and contrast enhancement (left) whereas brain metastases with absent PD-L1 expression had a more heterogenous texture and contrast enhancement (right). *MCC *maximum correlation coefficient; *ROI *region-of-interest

The most relevant features of the radiomics model (Fig. 3) comprised one histogram and two textural features and were used to define the radiomics signature. Besides the tumor volume, the three detected features showed significant or nearly significant differences in brain metastases with present or absent PD-L1 expression in both the training and the independent test set (Fig. 3). Representative local feature expression differences between presence and absence of PD-L1 expression in brain metastases are shown in Fig. 4.

## Discussion

The main finding of the present study suggest that a machine learning model based on radiomics features from routinely acquired structural MRI in combination with clinical parameters predicts the intracranial PD-L1 expression rate in NSCLC brain metastases with considerably high diagnostic accuracy. Of note, the diagnostic performance of the developed model could be confirmed in a small but independent test data set from another neurooncological center. Especially the external blinded validation, based on independent data using different scanners and imaging parameters, indicates the validity and robustness of the developed radiomics model suggesting that the model provides reliable results regardless of the imaging setup which increases its potential for a clinical translation.

Another main finding of our study is that we observed three particulars important radiomics features besides the contrast-enhancing tumor volume correlating with the PD-L1 expression in brain metastases secondary to NSCLC. The parameter coarseness extracted from the NGTDM, is a measure of the average intensity difference between the center voxel and its neighborhood, is an indicator of the spatial rate of change. A higher value indicates a lower spatial change rate and a locally more uniform texture. We observed higher coarseness in brain metastases expressing PD-L1, indicating a more uniform texture. In contrast, the parameter maximum correlation coefficient from the GLCM, as well as the histogram feature energy, showed higher values in brain metastases without PD-L1 expression reflecting a higher complexity of the texture. Accordingly, in our study, brain metastases without PD-L1 expression were larger and seemed to show a more complex texture and heterogenous pattern compared to brain metastases with PD-L1 expression (Fig. 4).

In patients with brain metastases secondary to NSCLC, recent findings indicate a discrepancy in extra- and intracranial PD-L1 expression and a heterogeneity in brain metastases in terms of the PD-L1 expression itself [13, 15, 29]. Accordingly, 15% of our patients showed a discrepancy between the intra- and extracranial PD-L1 expression. Similarly, differences in the tumor microenvironment in the brain may also be a predictive marker for response to immunotherapy [30, 31, 32]. Initial results showed that local immune features in patients with primary NSCLC might be reflected by characteristic radiological findings [33]. Especially for patients eligible for radiosurgery or fractionated radiotherapy as first-line therapy for brain metastases, tissue samples are usually not available. Thus, reliable tools for a non-invasive prediction of the intracranial PD-L1 expression and further development of radiomics signatures for characterization of the local immune features and quantification of PD-L1 expressing tumor-infiltrating lymphocytes are of high clinical relevance and may be beneficial for personalized precision treatment.

Radiomics models for the classification of subgroups matching the level of the intracranial PD-L1 expression (e.g., < 1%; 1–50%; >50%) may be of additional clinical value for the stratification of patients for immunotherapy using checkpoint inhibitors. Considering the small number of patients in our study, a further subgroup analysis would not provide meaningful results. Since the availability of tissue samples obtained from brain metastases independent of the primary tumor type for immunohistochemistry and other neuropathological analyses is generally still low, our initial results should be validated in a higher number of patients in a prospective multicenter setting enabling further subgroup analyses. Another limitation of our study design is that only T1 contrast-enhanced MRI was available for all patients in the training and test data. As multimodal and multisequence radiomics has shown potential to improve classification results, the value of including additional MR sequences and metabolic imaging using PET should be further evaluated in the future.

The usefulness of radiomics analysis for the evaluation of the PD-L1 expression rate in patients with NSCLC was already shown for the primary extracranial tumor. Wang et al. used radiomics features from chest CT scans for the classification of PD-L1 expression and EGFR mutation in primary NSCLC. Their developed deep-learning model achieved a good diagnostic performance with an AUC of 0.76 in the test data [34]. In other studies, a combination of clinical characteristics with CT radiomics features further improved the predictive performance for the PD-L1 expression (AUC of 0.85 and 0.84, respectively) [35]. In addition, metabolic imaging using 2-[^18^F]fluoro-2-deoxy-d-glucose PET in combination with CT features was used for radiomics model building with promising results for the prediction of PD-L1 expression [36].

As radiomics analyses provide additional information not accessible by conventional image analysis, such features appear to be of clinical value to serve as potential biomarkers for an improved tumor characterization. To enable a possible translation in clinical practice and broader acceptance of radiomics models, a better understanding of the biological meaning of radiomics features is crucial. Therefore, further prospective studies correlating imaging features with spatially correlated tissue samples and histomolecular work-up are needed.

## Conclusion

Our results suggest that the newly developed radiomics classifier allows a non-invasive prediction of the PD-L1 expression rate in patients with brain metastases secondary to NSCLC with considerably high diagnostic accuracy. Considering the discrepancies between the primary tumor and the brain metastases with respect to the PD-L1 expression rate, the model may be of clinical value for personalized treatment decisions, i.e., the administration of checkpoint inhibitor immunotherapy. Since our model is based on routinely acquired MR imaging data and the analysis lasts only a few minutes on a standard computer, it can be easily implemented in the clinical routine. Notwithstanding these promising results, further evaluation of the developed model, preferably in a higher number of patients, is needed.

## Data Availability

The datasets generated during and/or analyzed during the current study are available from the corresponding author on reasonable request.
